# Lowering the upper limit of serum alanine aminotransferase levels may reveal significant liver disease in the elderly

**DOI:** 10.1371/journal.pone.0212737

**Published:** 2019-04-11

**Authors:** Hemda Schmilovitz-Weiss, Rachel Gingold-Belfer, Alon Grossman, Nidal Issa, Doron Boltin, Yichayaou Beloosesky, Nira Morag Koren, Joseph Meyerovitch, Avraham Weiss

**Affiliations:** 1 Gastroenterology Unit, Rabin Medical Center, Hasharon Hospital, Petach Tikva, Israel; 2 Department of Surgery B, Rabin Medical Center, Hasharon Hospital, Petach Tikva, Israel; 3 Department of Gastroenterology, Rabin Medical Center, Beilinson Hospital, Petach Tikva, Sackler Faculty of Medicine, Tel Aviv University, Tel Aviv, Israel; 4 Department of Internal Medicine, Rabin Medical Center, Beilinson Hospital, Petach Tikva, Sackler Faculty of Medicine, Tel Aviv University, Tel Aviv, Israel; 5 Department of Geriatrics, Rabin Medical Center, Beilinson Hospital, Petach Tikva, Sackler Faculty of Medicine, Tel Aviv University, Tel Aviv, Israel; 6 Department of Epidemiology, Sackler Faculty of Medicine, Tel Aviv University, Tel Aviv, Israel; 7 Community Division, Clalit Health Services, Dan-Petach Tikva District, Petach Tikva Israel; University of Cape Town, SOUTH AFRICA

## Abstract

This study sought to determine the prevalence of significant liver disease in those subjects with serum alanine aminotransferase levels in the range between the current and the newly suggested upper limit of normal (termed the delta range). The files of the previous study subjects (who underwent at least one alanine aminotransferase measurement in 2002 and followed to 2012) were reviewed for a diagnosis of chronic liver disease; aspartate aminotransferase/platelet ratio index, FIB-4 and alanine aminotransferase/aspartate aminotransferase ratio were used to evaluate liver fibrosis. The prevalence of significant liver disease, by diagnoses and fibrosis scores was compared between subjects with alanine aminotransferase levels in the delta range (men, 42–45 IU/L; women, 26–34 IU/L) and in the newly suggested normal range (men, 15–42 IU/L; women, 10–26 IU/L). The cohort included 49,634 subjects (41% male, mean age 83±6 years) of whom 2022 were diagnosed with chronic liver disease including 366 with cirrhosis. Compared to subjects with alanine aminotransferase levels in the newly suggested normal range, subjects with alanine aminotransferase levels in the delta range had a significantly higher rate of chronic liver disease (men, 15.3% vs. 4.9%; women, 7.8% vs. 3.3%) and of cirrhosis specifically (men, 4.2% vs. 0.9%; women, 1.5% vs. 0.4%) and also had higher mean fibrosis scores (P <0.001 for all). Lowering the current upper limit of normal of serum alanine aminotransferase may help to identify elderly patients at risk of significant liver disease.

## Introduction

Higher levels of serum hepatocellular enzymes, alanine aminotransferase (ALT) and aspartate aminotransferase (AST), with or without higher levels of canalicular enzymes (alkaline phosphatase and gamma glutamyl transferase) and bilirubin, are a sign of significant liver disease. However, even an isolated mild elevation of serum ALT level above the normal range, may serve as a marker of liver injury [1,2] due to nonalcoholic fatty liver disease (NAFLD) and chronic hepatitis C infection (HCV) [3–6]. Furthermore, many studies have reported an association of elevated serum ALT level with increased liver-related morbidity and mortality [7,8] and all-cause mortality [9]. Recently, clinical guidelines have suggested lowering the upper limit of normal (ULN) of serum ALT for men and women [10–12]. This was supported by our previous study [12] showing that in elderly subjects, levels at the currently accepted ULN for ALT in Israel, were associated with significantly high mortality rates.

The aim of the present study was to investigate the prevalence of clinically significant liver disease in a large population of elderly subjects (age ≥65 years) residing in the community whose serum ALT levels fell within the range of the current ULN and the newly suggested ULN.

## Methods

Clalit Health Services (CHS) is the largest of the four health management organizations in Israel. Its member base numbers more than 4 million and 1.4 million in the Dan-Petach Tikva District Branch (in central Israel) alone. The comprehensive electronic data warehouse of CHS, aggregates continuous real-time medical input from hospital and community physicians and health service providers for each of its members.

The present study was performed on the same population as our previous one, which was derived from a review of the database of the CHS Dan-Petach Tikva District Branch. The study included community-dwelling subjects aged ≥65 years who had undergone at least one serum ALT measurement in 2002 and were followed until December 31, 2012. In our previous study, we queried the database for demographic data, co-morbidities, laboratory tests, and mortality [12].

The data of our patient records used in this retrospective study were fully anonymized: serial numbers were employed instead of patients’ names and ID# before we had any access to them. The study was approved by the CHS Ethics Committee (approval no. 0007-12-COM).

Serum ALT levels were measured with a Beckman Coulter AU analyzer using a quantitative kinetic UV method, as recommended by the International Federation of Clinical Chemistry, but with the substitution of pyridoxal-5’-phosphate with L-alanine and 2-oxoglutarate as substrates [13]. The prevalence of liver disease in this study was evaluated according to the diagnoses in the medical records. Additionally, we applied three established surrogate methods that measure the degree of liver fibrosis [14]: AST-to-platelet ratio index (APRI) [15], FIB-4 score [16], and ALT/AST ratio (AAR) [17] The APRI is calculated using the formula: AST level (IU/L)/AST (upper limit of normal) (IU/L)/ platelet count (10^9^/L) x 100. The FIB-4 is calculated using the formula: age (years) x AST level (IU/L)/platelet count (10^9^/L) x √ ALT (IU/L). The AAR was estimated by dividing the AST level by the ALT level. Clinically significant liver disease is usually associated with advanced fibrosis (F3) [18–20]; liver cirrhosis is characterized by the highest stage of fibrosis (F4) [21].

CHS defines the normal range of serum ALT as 0–34 IU/L for women and 0–45 IU/L for men, based on the Clinical Guide to Laboratory Tests [22–24]. Our previous analysis of the association of serum ALT level with mortality rates suggested that a new range of 15–42 IU/L for men and 10–26 IU/L for women be established [12]. For the present study, we identified the individuals within this population whose serum ALT levels fell between the accepted ULN and the newly suggested ULN range, which we termed the delta range (42–45 IU/L for men, 26–34 IU/L for women). The prevalence of CLD and liver cirrhosis was compared between this group and the remaining subjects, overall and by sex.

### Statistical analysis

Data are presented as mean and standard deviation for continuous variables and frequency and percentage for categorical variables. Data were analyzed with the SPSS, version 23.0 (SPSS Inc., Chicago, IL, USA). The level of significance was set at 0.05. Chi-square tests and independent t-tests compared categorical and continuous variables, respectively, between groups (CLD/no CLD, cirrhosis/no cirrhosis). CLD and cirrhosis prevalence rates were compared by ALT categories using chi-square tests. Mean fibrosis test values were compared by ALT categories using analysis of variance followed by the Bonferroni post hoc test. Independent t-tests compared mean laboratory and fibrosis test values between groups (CLD/no CLD, cirrhosis/no cirrhosis). The receiver operating characteristic (ROC) curve ploted the 1-specificity against the sensitivity of each possible cut-point on the continuum of the predictor variable. In the present study, the ability of the model to predict cirrhosis was evaluated using the area under the ROC curve (AUC).

## Results

The study included 49,634 subjects, 41% male, median age 83±6 years (range: 65–115 years).

CLD was diagnosed in 2022 subjects of whom 366 had cirrhosis. CLDs included NAFLD, chronic HCV, chronic hepatitis B infection (HBV), liver cirrhosis of any etiology and other liver diseases of various etiologies (alcoholic cirrhosis, cryptogenic cirrhosis, sarcoidosis, amyloidosis). NAFLD was the most common etiology for CLD (77.8%) [12].

Men had a significantly higher prevalence than women of CLD (4.3% vs 3.9%, P = 0.047) and of liver cirrhosis (0.9% vs 0.6%, P<0.001).

Compared to subjects without CLD, the subjects with CLD (all types) had significantly higher prevalence rates of some diseases related to the metabolic syndrome (i.e., diabetes mellitus, hypertension and borderline higher prevalence of ischemic heart disease) and higher rates of malignancies (Table 1). Other significant findings were a lower all-cause mortality rate, higher mean hemoglobin concentration, higher mean serum albumin level, and lower mean cholesterol level (Table 1). Subjects with liver cirrhosis had significantly higher malignancy rates than subjects without liver cirrhosis in addition to a significantly higher all-cause mortality rate and lower mean values of hemoglobin concentration, serum albumin, and cholesterol (Table 1).

**Table 1:** Baseline characteristics, associated diseases and laboratory results by presence of CLD and cirrhosis (n = 49634).

Parameters	CLD n = 2022	No CLD n = 47612	*P*	Cirrhosis n = 366	No cirrhosis n = 49268	*P*
Age (yr), mean±SD	81±5.2	83±6.4	<0.001	81±6.1	83±6.3	<0.001
Male gender, n (%)	878 (43)	19615 (41)	0.47	179 (48)	20314 (41)	0.008
IHD, n (%)	889 (44)	19958 (42)	0.068	168 (45)	20679 (42)	0.233
CHF, n (%)	458 (23)	10364 (22)	0.346	103 (28)	10719 (22)	0.006
CRF, n (%)	463 (23)	9331 (20)	<0.001	111 (30)	9683 (20)	<0.001
Diabetes mellitus, n (%)	864 (43)	15574 (33)	<0.001	147 (39)	16291 (33)	0.010
Hypertension, n (%)	1622 (80)	35751 (75)	<0.001	283 (76)	37090 (75)	0.796
CVA/TIA, n (%)	459 (23)	11831 (25)	0.028	75 (20)	12215 (25)	0.037
Malignancy, n (%)	327 (16)	6182 (13)	<0.001	91 (24)	6418 (13)	<0.001
Death (all-cause), n (%)	687 (34)	21377 (45)	<0.001	201 (54)	21863 (44)	<0.001
Hemoglobin (gr/dL), mean±SD	14.0±1.4	13.7±1.4	<0.001	13.7±1.5	13.8±1.4	0.842
WBC (K/μL),mean±SD	7.87±3.2	8.28±3.9	<0.001	7.59±3.0	8.27±3.9	0.001
Albumin (gr/dL), mean±SD	4.05±0.38	4.01±0.38	<0.001	3.92±0.43	4.02±0.38	<0.001
Total cholesterol (mg/dL), mean±SD	200±42	208±41	<0.001	189±45	207±41	<0.001
Bilirubin (mg/dL), mean±SD	0.43±1.1	0.36±0.9	0.003	0.76±1.8	0.36±0.94	<0.001
CRP (mg/dL), mean±SD	2.22±7.9	2.95±11.0	0.003	2.29±5.1	2.98±10.9	0.439
TSH (mIU/L), mean±SD	2.60±2.35	2.66±4.6	0.579	2.64±2.4	2.66±4.5	0.932

CLD, chronic liver disease; IHD, ischemic heart disease; CHF, congestive heart failure; CRF, chronic renal failure; CVA, cerebrovascular accident; TIA, transient ischemic attack; WBC, white blood count; CRP, C-reactive protein; TSH, thyroid-stimulating hormone

ALT levels were evaluated separately for women and men. Among the women, those with an ALT level within the delta range had a threefold higher prevalence of cirrhosis and a twofold higher prevalence of CLD than women with an ALT level within the lower, newly suggested normal range (cirrhosis, 1.5% vs. 0.4%; CLD, 7.8% vs. 3.3%) (Table 2). The same comparison in men yielded a fourfold higher prevalence of cirrhosis and a threefold higher prevalence of CLD in those with delta-range ALT levels than in those with ALT levels within the newly suggested range (cirrhosis, 4.2% vs. 0.9%; CLD, 15.3% vs. 4.9%) (Table 2).

**Table 2:** Prevalence of cirrhosis and CLD by ALT categories in women and men.

Liver disease	ALT below normal	ALT suggested normal	ALT delta range*	ALT above normal	*P*^†^
Women					
ALT range (IU/L)	0–10	10–25	26–34	34+	
No. subjects	4708	21132	1887	1414	
CLD, n(%)	70 (1.5)	689 (3.3)	147 (7.8)	238 (16.8)	<0.001
Cirrhosis, n(%)	6 (0.1)	78 (0.4)	28 (1.5)	68 (4.8)	<0.001
Men					
ALT level (IU/L)	0–15	15–42	42–45	45+	
No. subjects	8315	11401	118	659	
CLD, n(%)	185 (2.2)	553 (4.9)	18 (15.3)	122 (18.5)	<0.001
Cirrhosis, n(%)	35 (0.4)	105 (0.9)	5 (4.2)	41 (6.2)	<0.001

ALT, alanine aminotransferase; CLD, chronic liver disease

*Delta range- range of ALT values between the current and suggested upper limit of normal

^†^Differences in the prevalence rates of CLD and cirrhosis between subjects with values in the delta range and subjects with values within the newly suggested range (lower ULN) were statistically significant.

**Fig 1:**
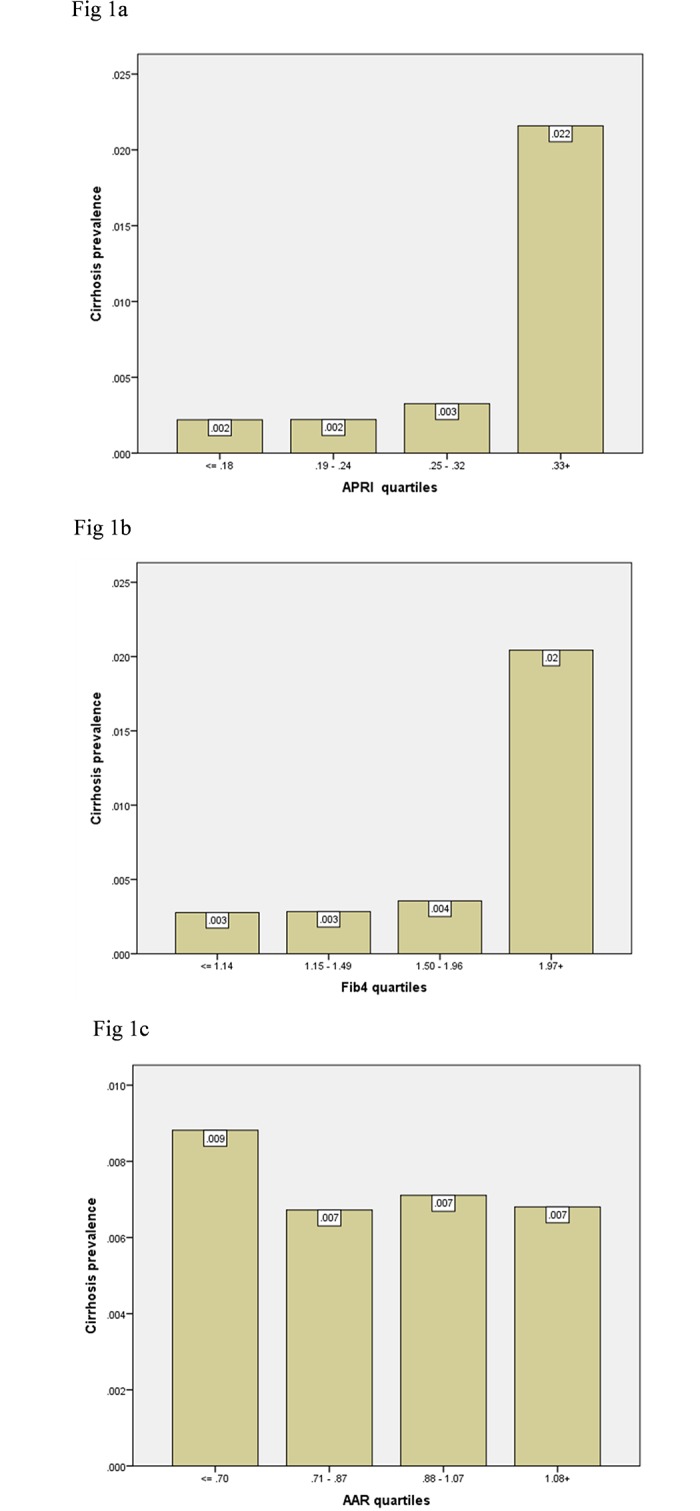
a—cirrhosis prevalence by quartiles of fibrosis test results by APRI; b- cirrhosis prevalence by quartiles of fibrosis test results by FIB-4; c—cirrhosis prevalence by quartiles of fibrosis test results by AAR.

The prevalence rates of cirrhosis by quartiles of the APRI, FIB-4, and AAR results are presented in Fig 1a, 1b and 1c (note that the highest frequencies of cirrhosis are associated with the upper quartile of APRI and FIB-4 values).

Mean values on all three tests were significantly higher in the subjects (men and women) with ALT levels within the delta range than in subjects with levels within the newly suggested normal range (Table 3).

**Table 3:** Mean scores on fibrosis tests by ALT categories in women and men.

Fibrosis tests	ALT below normal	ALT suggested normal	ALT delta range*	ALT above normal	*P*^†^
Women					
ALT level (IU/L)	0–10	10–25	26–34	34+	
No. subjects	4708	21132	1887	1414	
FIB-4, mean±SD	1.7±0.9	1.55±1.2	1.66±1.0	2.46±2.5	<0.001
APRI, mean±SD	0.20± 0.1	0.26±0.2	0.38±0.2	0.89±1.3	<0.001
AAR, mean±SD	0.61±0.2	0.88±0.2	1.15±0.3	1.40±1.2	<0.001
Men					
ALT level (IU/L)	0–15	15–42	42–45	45+	
No. subjects	8315	11401	118	659	
FIB-4	1.8±1.0	1.8±1.2	2.2±2.2	3.5±4.8	<0.001
APRI	0.23±0.1	0.31±0.2	0.57±0.6	1.26 ±0.6	<0.001
AAR	0.76±0.2	1.05±0.3	1.34±0.5	1.60±2.4	<0.001

AAR, aspartate aminotransferase-to-alanine aminotransferase ratio; ALT, alanine aminotransferase; FIB-4, fibrosis 4; APRI, aspartate aminotransferase (AST)-to-platelet ratio index; AAR, ALT/AST ratio

*Delta range—range of ALT values between the current and suggested upper limit of normal

^†^Differences in mean fibrosis scores between subjects with values in the delta range and subjects with values within the newly suggested range (lower ULN) were statistically significant.

**Fig 2:**
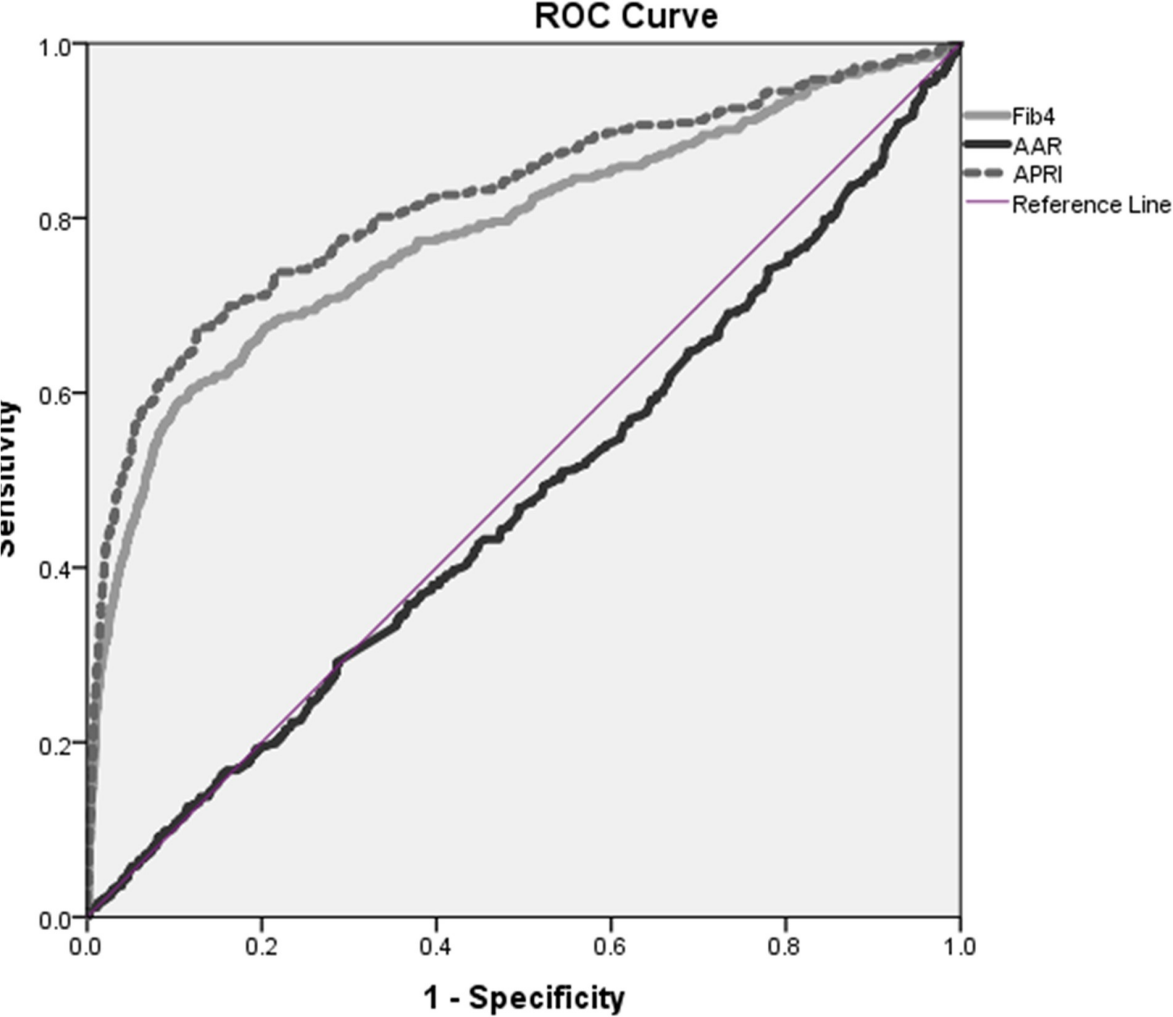
Area under the curve for results of the three fibrosis tests.

Fig 2 shows the results of the ROC curve analysis of the power of the fibrosis scores to predict cirrhosis. The AUC was 0.821 for the APRI and 0.78 for the FIB-4. However, owing to their low sensitivity and specificity, these tests cannot serve as screening tools for cirrhosis.

Subjects with CLD or cirrhosis had significantly higher ALT and AST levels and a significantly lower platelet count than subjects without liver disease. The APRI and FIB-4 fibrosis scores were significantly higher in the subjects with CLD, and specifically the cirrhotic patients, whereas the AAR score was significantly higher only in the subjects with CLD (Table 4).

**Table 4:** Laboratory and fibrosis test results for CLD and cirrhosis.

Diagnostic tests	CLD N = 2022	No CLD N = 47612	*P*	Cirrhosis N = 366	No cirrhosis N = 49268	*P*
ALT	29.4±31.8	19.0±45.3	<0.001	40.4±55.7	19.3±44.8	<0.001
AST	30.0±29.3	21.2±20.1	<0.001	41.4±41.1	21.4±20.4	<0.001
PLT	229±77	248±78	<0.001	202±89	248±77	<0.001
FIB-4	2.22±2.9	1.69±1.2	<0.001	3.4±3.8	1.7±1.3	<0.001
APRI	0.54±1.07	0.29±0.33	<0.001	1.00±1.8	0.29±0.36	<0.001
AAR	1.00±0.47	0.91±0.46	<0.001	0.95±0.47	0.91±0.46	0.107

All values are mean±SD.

ALT, alanine aminotransaminase; AST, aspartate aminotransaminase; PLT, platelet count; FIB-4, fibrosis 4 test; APRI, AST-to-platelet ratio index; AAR, ALT/AST ratio

## Discussion

Our earlier study suggested that the accepted ULN for serum ALT level should be lowered in the elderly population, to 10–26 IU/L for women and 15–42 IU/L for men [12]. In the present study, we tested the value of lowering the ULN for ALT for the detection of liver disease. Using the same large health management organization registry of community dwelling elderly subjects, we found, on the basis of the clinical diagnoses and the estimated fibrosis scores, that significant liver disease was more prevalent in individuals with ALT levels in the delta range between the current and suggested ULN than in individuals with levels below the newly suggested ULN. This finding indicates that elderly individuals who meet the currently accepted normal range of ALT may in fact harbor clinically significant liver disease.

The higher mean hemoglobin concentration and serum albumin level observed in this study in the subjects with CLD relative to the subjects without CLD might be attributed to their higher prevalence of NAFLD; that is, they were probably well fed with a good nutritional status. Their mean serum cholesterol level, however, was lower than that of the rest of the cohort. It is possible that because of the greater frequency of cardiovascular risk factors in the subjects with CLD, they received better health care than the subjects without CLD, including cholesterol-lowering drugs. Notably, although the between-group differences (CLD/no CLD and cirrhosis/no cirrhosis) in many laboratory parameters, mainly hemoglobin, albumin, and cholesterol, were statistically significant, the actual differences were relatively small, apparently because of the difference in group size.

NAFLD, chronic HCV, and chronic HBV are the main causes of hepatocellular carcinoma [25–27]. HCV and HBV are also associated with other malignancies [28,29]. The higher malignancy rates found in the whole CLD group and the cirrhotic subgroup in our study might be attributed to these factors. In the advanced stages of CLD, serum ALT level loses its prime diagnostic importance to other clinical and laboratory findings. However, in the early stages of the disease, abnormal ALT levels may raise the index of suspicion of CLD among clinicians, leading to earlier diagnosis and treatment [30,31]. Thus, lowering the ULN of serum ALT may help to identify a greater proportion of patients with significant liver disease. Early diagnosis and work-up is particularly important today, given the new medical technologies and medications directed at safely eradicating HCV [32] and halting the progression of chronic severe HBV (nucleoside/nucleotide analogues) [33] and possibly NAFLD [34,35]. Additionally, liver cirrhosis is associated with a high risk of morbidity and mortality from liver insufficiency or hepatocellular carcinoma [36,37], and its early detection can lead to curative treatment [38].

The most prevalent occult CLD in the elderly is probably NAFLD [39]. NAFLD represents the expression of the metabolic syndrome in the liver [40]. The associated co-morbidities of the metabolic syndrome, namely arterial hypertension, diabetes mellitus, hyperlipidemia, ischemic heart disease, and stroke, are much more prevalent in the elderly [41]. Thus, following a primary diagnosis of NAFLD, patients must be made aware of the necessity for life-style changes and regular monitoring of serum glucose, lipid levels, and blood pressure. In patients considered to have a normal ALT level, the significance of an imaging (ultrasound, computed tomography) finding of fatty infiltration of the liver would probably be underestimated, and management measures would not be instituted. Under these circumstances using a lower ULN for ALT may aid clinicians in the earlier diagnosis of NAFLD.

Hepatic encephalopathy is a common complication of cirrhosis which results in poor brain functioning. It ranges from clinically minimal to clinically overt brain dysfunction [42]. In the elderly, minimal encephalopathy may be mistaken for dementia. Therefore, its early diagnosis is important to avoid erroneous and unnecessary work-up and treatments [43]. Drug-induced liver injury (DILI) is common in the elderly owing to their high rates of polypharmacy [44]. DILI usually presents as a high serum ALT level (~500 IU/L) [45], although in some patients, such as those using acetaminophen in the therapeutic range, liver enzymes are just mildly elevated [46]. Clinicians must be made aware that even a slight deviation in serum ALT above the ULN may indicate liver injury and that elimination of the offending agent might prevent ongoing injury [47].

Our findings are in accordance with the recent American College of Gastroenterology (ACG) guidelines [6] which recommend clinical evaluation for liver disease in healthy men with a serum ALT level above 33 IU/L and in women with a serum ALT level above 25 IU/L. The slight differences between the ULN values used here, which were derived from our earlier study [12] and the ACG recommendations may be at least partly explained by age differences in the populations evaluated. The ACG guidelines are based on a meta-analysis of studies of adults of various ages whereas we focused exclusively on the over-65-year age group. Older adults are more prone to hepatic injury due to age-associated reductions in hepatic regenerative capacity, functional hepatocyte volume, and hepatic blood flow [48].

The APRI, FIB-4, and AAR scores have been found to have a good correlation with significant liver fibrosis. Indeed, this was also found in our study mainly for the FIB-4 and APRI scores but not for AAR scores (Fig 2). These fibrosis scores have an inherent drawback: the higher the sensitivity in detecting liver fibrosis, the lower the specificity and vice versa [14–17]. The higher fibrosis scores in the subjects with ALT levels in the delta range in our study group compared to those with lower levels further support the need to apply the newly suggested ULN to the elderly, in Israel and possibly worldwide.

### Study limitations

Our study is limited by the biases inherent in a retrospective design. This study was based on a large registry of community dwelling subjects attending the largest health management organization in Israel (CHS), and the results are in accordance with the global literature. However, the catchment area of the Dan-Petach Tikva District Branch of CHS consists almost entirely of a Jewish population, which may limit the generalizability of our findings.

To the best of our knowledge, there are no previous studies of the significance of the ALT delta range to the diagnosis of significant liver disease in the elderly.

## Conclusions

Lowering the ULN of serum ALT in the elderly may help to identify patients with significant liver disease. A global standardization of this simple, low cost, and highly available laboratory test is needed and may upgrade the standard of care for these individuals worldwide.

## Supporting information

S1 Dataset Basic characteristics of the study cohort. (XLSX)
